# An equity analysis of utilization of health services in Afghanistan using a national household survey

**DOI:** 10.1186/s12889-016-3894-z

**Published:** 2016-12-05

**Authors:** Christine Kim, Khwaja Mir Ahad Saeed, Ahmad Shah Salehi, Wu Zeng

**Affiliations:** 1 Gillings School of Global Public Health, University of North Carolina at Chapel Hill, Chapel Hill, NC 27599 USA; 2 Ministry of Public Health, Kabul, Afghanistan; 3 Ministry of Labour and Social Affairs, Kabul, Afghanistan; 4 Schneider Institutes for Health Policy, Heller School, Brandeis University, MS 035, Waltham, MA 02454 USA

## Abstract

**Background:**

Afghanistan has made great strides in the coverage of health services across the country but coverage of key indicators remains low nationally and whether the poorest households are accessing these services is not well understood.

**Methods:**

We analyzed the Afghanistan Mortality Survey 2010 on utilization of inpatient and outpatient care, institutional delivery and antenatal care by wealth quintiles. Concentration indexes (CIs) were generated to measure the inequality of using the four services. Additional analyses were conducted to examine factors that explain the health inequalities (e.g. age, gender, education and residence).

**Results:**

Among households reporting utilization of health services, public health facilities were used more often for inpatient care, while they were used less for outpatient care. Overall, the utilization of inpatient and outpatient care, and antenatal care was equally distributed among income groups, with CIs of 0.04, 0.03 and 0.08, respectively. However, the poor used more public facilities while the wealthy used more private facilities. There was a substantial inequality in the use of institutional delivery services, with a CI of 0.31. Poorer women had a lower rate of institutional deliveries overall, in both public and private facilities, compared to the wealthy. Location was an important factor in explaining the inequality in the use of health services.

**Conclusions:**

The large gap between the rich and poor in access to and utilization of key maternal services, such as institutional delivery, may be a central factor to the high rates of maternal mortality and morbidity and impedes efforts to make progress toward universal health coverage. While poorer households use public health services more often, the use of public facilities for outpatient visits remains half that of private facilities. Pro-poor targeting as well as a better understanding of the private sector’s role in increasing equitable coverage of maternal health services is needed. Equity-oriented approaches in health should be prioritized to promote more inclusive health system reforms.

## Background

The first Universal Health Coverage (UHC) day was held in December 2014, recognizing the right for all people to access quality, essential health services when needed without financial hardship [1]. The concept of UHC was first globally promoted through the 2010 World Health Report, but progress toward achieving UHC requires the inclusion of vulnerable populations and targeted health system planning for greater equity [1, 2]. Following the Millennium Development Goals (MDG) in 2015, the expanded Sustainable Development Goals (SDG) will introduce a more ambitious health agenda including UHC [3]. Health equity or bridging the disparities between rich and poor access to quality health services is central to meeting this agenda. Yet making health systems equitable continues to be a challenge in global health.

After decades of conflict, the Government of Afghanistan began implementing the Basic Package of Health Services (BPHS) in 2003 to provide a standardized package of basic primary health care services across the country [4]. This was complemented by the Essential Package of Hospital Services (EPHS), introduced in 2005, to increase referrals and access to hospital services [5]. Partially as a result of the BPHS and EPHS program, the health of the population has dramatically improved since the rebuilding of the country’s health system. Infant mortality decreased to 45 for the period of 2006–2010 from 66 deaths per 1,000 births for the period of 2001–2005, under-5 mortality is down to 55 from 87 per 1,000 live births during the same time frame, and maternal mortality ratio was estimated at 327 per 100,000 live births in 2010 [6–9]. These rates remain high, compared to the global average of infant mortality of 32 per 1,000 live births, of under 5 mortality of 43 per 1,000 live births, and of maternal mortality of 216 per 100,000 live births in 2015 [10, 11]. Despite this progress, only 45.7% of children aged 12–23 months are fully vaccinated, 55% of children under five years suffer from stunting, and only 48.1% of women deliver in a health facility [9, 12].

The BPHS and EPHS expanded access to primary and secondary care services, now covering about 57% of the population according to the most recent estimates [13]. Public health services are delivered through “contracting-out” mechanisms by which non-governmental organizations (NGO) deliver a set of services as defined by the BPHS and EPHS [4, 5, 14]. Studies have shown that contracting mechanisms in low-income settings can help improve equitable service delivery [14]. Yet, less is understood about the reach and quality of the private health sector in Afghanistan. While the delivery of health services through public facilities has expanded, since 2006, almost 60% of the population reported obtaining services from private providers such as private hospitals, clinics, pharmacies, and doctor’s offices [15, 16]. As the demand for health services increases, the government aims to build stronger partnerships with the private health sector to bridge the gap in the delivery of quality services through improved regulations and establishment of minimum required standards [17].

The challenges to Afghanistan’s health system and health financing, coupled with the advocacy for UHC, call for particular attention vulnerable populations such as women and children. This in turn requires a better understanding of the distribution and inequality of health service delivery. Previous equity analyses have explored the distribution of public health service use by contracting mechanism [14], and for specific populations such as people with disabilities [18, 19]. This paper explores the equity in utilization of health services among different populations compared to previous studies and among users of both public and private health services.

Understanding equity in the utilization of health services in both the public and private sectors contributes to a more holistic perspective of population access and use of care. This paper analyzes both inequality and inequity of inpatient care, outpatient care, and maternal health care services in public health facilities compared to private health facilities in Afghanistan, to help understand how public and private health services are utilized across the population.

## Methods

Measuring and operationalizing inequity and inequality in the health system seem ambiguous as they are more often perceived as human rights or ethical principles [20]. But significant efforts have been made to define, frame, and measure inequity and inequality using more unified approaches [21–23]. Regarding inequality and inequity of utilization of health services, inequality is defined as any differences in health utilization between different population groups, while inequity is the part of inequality that is considered unjustified, where factors correlated with health are considered unfair due to the inability to access an equal amount of care based on need regardless of socioeconomic status [21].

To conduct the inequality and inequity analysis of utilization of health services, information on socioeconomic status, utilization of health services (inpatient care vs. outpatient care), and other factors that explain the inequality (e.g. age, gender, education and residence [Urban vs. rural]) was extracted from a national household survey, the Afghanistan Mortality Survey (AMS) 2010 [6]. The AMS was a multi-stage stratified sample of enumeration areas then households, with selection by probability proportional to size sampling. Stratification was achieved by zone (North, Central, and South) and then by residence type (urban and rural). Due to security reasons, three provinces were excluded from the southern zone. In each zone, 87% of the population was covered, but in the southern zone, only 66% was covered. The AMS included 22,351 households, with a total sample size of 180,676 in its original dataset, of which 50.7% were males and 49.3% were females [6].

Measurement of socioeconomic status of a household: We used the constructed wealth status measurement by the AMS. The wealth of a household was measured by assessing household assets (e.g. the ownership of a television and bicycle); and, household dwelling characteristics (e.g. source of drinking water, sanitation facilities, and dwelling construction material of floors and roofs). A weight generated from principal component analysis was assigned for each asset, and was used to generate an asset score, which was normalized with a mean of zero and standard deviation of one. A wealth index was constructed as the sum of the scores for all the assets in the household. More detailed information on construction of the wealth index can be found in the AMS 2010 report [6].

Measurement of utilization of health services: Heads of households were asked about the utilization of general inpatient care, and outpatient care for all household members, while women who had at least one child in the last five years at the time of survey reported utilization of maternal health services and child health services. The key indicators included in this analysis are institutional delivery with a skilled birth attendant and any antenatal care (ANC) used during the last pregnancy for women who had one or more births in the last five years at the time of the survey. General utilization indicators included are inpatient admissions in the last 12 months and outpatient visits in the last month. Institutional delivery, any ANC, and having an inpatient admission or and outpatient visit were all measured as binary variables. Respondents were also asked about where services were obtained for their last visit (or admission), allowing analysis of service utilization by health facility type at public facilities (e.g. national hospital, regional hospitals, provincial hospitals, district hospitals, poly clinics and other public clinical units) or private facilities (e.g. private hospitals, private clinics and other private clinical units). Depending on where the services were provided, the use of inpatient and outpatient care was categorized as those provided at public or private facilities. Singling out services at public facilities from private facilities would help understand the inequality and inequity of the use of key services in different settings. The original dataset of AMS contained 22,351 households with 180,676 individuals. Most data were complete and no multiple imputation was conducted. When implementing the analysis, we removed 705 cases that were reported the use of any of inpatient and outpatient care and maternal care, but could not be linked to the master individual level dataset (180,676 individuals). This accounted for a 1.4% reduction of cases using the care under the analysis (705 out of 51,024). Additionally, there were 464 cases missing the information on education and 38 cases missing the information on gender. As a result, a total number of 180,012 individuals were included for analyzing utilization of outpatient and inpatient care. When analyzing delivery and ANC, we removed 6,663 households that did not have such occurrences, which left 15,688 households with 18,255 pregnant women in the analysis.

In this study, we first present the analysis of health service utilization by wealth quintile, residence, gender, and other individual characteristics. Then we present the concentration index measuring the inequality and its decomposition by both individual (e.g. age and gender) and household (e.g. residence) characteristics for each of the indicators included in the analysis that provides estimates of inequities. We then provide the analysis of utilization of target services at public health facilities, including BPHS or EPHS.

The descriptive analysis was conducted using Stata [24], with national population weights. Analysis of inequality and inequity was conducted by using the ADePT software tool developed by the World Bank [25]. We used age and gender as standardizing variables (gender was omitted when analyzing ANC and institutional delivery), and residence and education as control variables when implementing the analysis using ADePT. The weight of households were specified and were taken into consideration when estimating inequality and inequity. The ADePT software tool automates economic analysis techniques for user countries on health and economic impacts, poverty, and inequality. The main equity and inequality outputs from the tool include concentration indexes, concentration curves, and decomposition of the concentration indexes. The tool calculates concentration indexes following the procedures described in O’Donnell et al. (2007) for micro-data, that is, when values for health variables and previously constructed wealth measures are available for each individual observation [26]. The concentration index is a measure of inequality that is estimated as the transformation of a variable of interest on fractional rank of wealth for a population. It ranges from −1 to 1, i.e. from a perfect pro-poor distribution to a pro-rich distribution. The concentration curve displays the share of health variable accounted for by cumulative proportions of individuals in the population ranked from poorest to richest. Lastly, the decomposition distinguishes the inequality measure from inequity by both “need” and “non-need” factors. The decomposition could be represented by stacked bars. Each factor is drawn above or below the horizontal line at zero in the stacked bar– above the line indicates a positive contribution of the factor making the concentration index more pro-rich and below the line indicates a negative contribution of the factor making the concentration more pro-poor. The larger the area of the variable in the graph, the greater its contribution to making the concentration index more pro-rich or pro-poor. The residuals show the part of the concentration index that is not due to the factors included in the analysis. In this study, we regard gender and age as “need” variables that predict the need for health services, while regard education and residence as “non-need” variables, from which the differences of utilization resulted are considered as unfair and as inequity.

## Results

The demographic characteristics of women and the general population from the national household survey are shown in Table 1. The mean age of women in the sample was 29.1 years; Women aged between 26 to 30 years old accounted for the largest share of the sample (26.40%); 88.2% of women had no education and 81.1% were from rural areas. In the general population, analyzed for inpatient and outpatient service utilization, men were 50.9% of the population. The mean age of the general population in the sample was 28.7 years, ranging from 0 to 95 years old; the largest age group came from the under 10 years old, accounting for 31.9%. A vast majority (80.0%) lived in rural areas.

**Table 1:** Demographic characteristics

Mean ± SD/percentage			
Pregnant women^a^		General population	
Age	29.11 ± 0.06	Age	28.71 ± 0.06
14–19	5.05%	0–10	31.88%
20–25	23.72%	11–20	27.22%
26–30	26.40%	21–30	15.52%
30–35	18.62%	31–40	9.26%
35–40	15.25%	41–50	6.76%
40–45	7.76%	51–60	4.79%
45–49	3.21%	60+	4.57%
Gender		Gender	
Male	0.00%	Male	50.98%
Female	100.00%	Female	49.02%
Urban		Urban	
Non-urban	81.10%	Non-urban	80.02%
Urban	18.90%	Urban	19.98%
Education		Education	
No education	88.23%	No education	87.96%
Some education	11.77%	Some education	12.04%

^a^Pregnant women refer to women who had at least one child in the last five years at the time of the survey

Table 2 shows the mean use of outpatient and inpatient services by individual and household characteristics. Households used private health facilities more for outpatient care (12.7% vs. 6.7%; *p* < 0.001) and public health facilities more for inpatient care (3.3% vs. 0.7%; *p* <0.001). Households with some education had statistically significant higher utilization of services compared to households with no education for both outpatient and inpatient care (*p* < 0.001) in all settings except for public facilities for outpatient care where there was a similar trend but not a significant difference. Similarly, the poorest households had the least utilization of outpatient and inpatient care compared to the wealthiest households (*p* < 0.001). Utilization of both outpatient and inpatient services were higher among males compared to females for both inpatient and outpatient care in both public and private settings (*p* < 0.001). Utilization increased by age group with similar trends in use for private health facilities for outpatient care and public health facilities for inpatient care.

**Table 2:** Utilization of outpatient and inpatient care by household characteristics

	Outpatient care (Mean ± Std. Err.)			Inpatient care (Mean ± Std. Err.)		
	All	Public	Private	All	Public	Private
Residency						
Urban	0.231 ± 0.0019	0.040 ± 0.0009	0.187 ± 0.0017	0.045 ± 0.0009	0.034 ± 0.0008	0.010 ± 0.0004
Rural	0.190 ± 0.0012	0.074 ± 0.0008	0.112 ± 0.0010	0.042 ± 0.0006	0.033 ± 0.0006	0.007 ± 0.0003
t value	17.86***	−27.35***	36.88***	3.01**	0.81	5.59***
Education						
No education	0.244 ± 0.0038	0.079 ± 0.0025	0.161 ± 0.0032	0.057 ± 0.0020	0.045 ± 0.0019	0.009 ± 0.0008
Some education	0.353 ± 0.0109	0.089 ± 0.0069	0.261 ± 0.0097	0.102 ± 0.0070	0.075 ± 0.0060	0.023 ± 0.0038
t value	9.39***	1.36	9.69***	5.98***	4.75***	3.42***
Gender						
Male	0.234 ± 0.0016	0.080 ± 0.0011	0.150 ± 0.0013	0.067 ± 0.0009	0.053 ± 0.0008	0.011 ± 0.0004
Female	0.161 ± 0.0014	0.054 ± 0.0009	0.104 ± 0.0011	0.017 ± 0.0005	0.013 ± 0.0004	0.003 ± 0.0002
t value	34.16***	18.34***	26.48***	46.81***	41.84***	18.06***
Wealth^∆^						
1st quintile	0.169 ± 0.0025	0.086 ± 0.0018	0.080 ± 0.0018	0.038 ± 0.0012	0.032 ± 0.0011	0.005 ± 0.0005
2nd quintile	0.200 ± 0.0026	0.082 ± 0.0018	0.115 ± 0.0020	0.043 ± 0.0013	0.035 ± 0.0012	0.006 ± 0.0005
3rd quintile	0.191 ± 0.0024	0.062 ± 0.0015	0.124 ± 0.0020	0.040 ± 0.0012	0.033 ± 0.0011	0.006 ± 0.0005
4th quintile	0.204 ± 0.0023	0.064 ± 0.0015	0.135 ± 0.0019	0.044 ± 0.0011	0.034 ± 0.0010	0.008 ± 0.0005
5th quintile	0.220 ± 0.0020	0.048 ± 0.0011	0.169 ± 0.0018	0.045 ± 0.0010	0.033 ± 0.0009	0.011 ± 0.0005
*F* value	60.16***	176.17***	343.56***	6.24***	2.43*	23.38***
Age						
0–10	0.034 ± 0.0008	0.010 ± 0.0005	0.024 ± 0.0007	0.001 ± 0.0002	0.001 ± 0.0001	0.000 ± 0.0001
11–20	0.075 ± 0.0013	0.024 ± 0.0008	0.050 ± 0.0011	0.005 ± 0.0004	0.004 ± 0.0003	0.001 ± 0.0002
21–30	0.242 ± 0.0029	0.081 ± 0.0019	0.158 ± 0.0024	0.043 ± 0.0014	0.035 ± 0.0013	0.008 ± 0.0007
31–40	0.470 ± 0.0044	0.165 ± 0.0035	0.299 ± 0.0040	0.099 ± 0.0026	0.083 ± 0.0024	0.016 ± 0.0010
41–50	0.574 ± 0.0051	0.208 ± 0.0044	0.360 ± 0.0049	0.148 ± 0.0037	0.121 ± 0.0034	0.027 ± 0.0016
51–60	0.520 ± 0.0062	0.181 ± 0.0050	0.335 ± 0.0058	0.137 ± 0.0042	0.111 ± 0.0039	0.026 ± 0.0019
60+	0.438 ± 0.0062	0.154 ± 0.0047	0.280 ± 0.0054	0.139 ± 0.0043	0.116 ± 0.0040	0.023 ± 0.0018
*F* value	5397.41***	1179.11***	2674.39***	1028.42***	793.35***	155.82***
Total	0.198 ± 0.0010	0.067 ± 0.0007	0.127 ± 0.0008	0.042 ± 0.0005	0.033 ± 0.0005	0.007 ± 0.0002

∆1st quintile are the poorest households, 5th quintile are the richest households; Std. Err. Denotes standard error; * *p* < 0.05, **p < 0.01, ***p < 0.001; All numbers are weighted by household sampling weights; Due to rounding errors and missing values, the numbers for public and private facilities do not perfectly add to the numbers for all facilities

Utilization of ANC and institutional delivery is shown in Table 3. A higher percentage of women residing in urban areas used ANC and institutional delivery. 57% of women in rural areas used ANC and 24.5% used institutional delivery, whereas 85.4% of urban women used ANC and 65.2% used institutional delivery, and these differences are statistically significant (*p* < 0.001). Women in urban areas were more likely to use private health facilities for ANC services (55.6%) but to deliver in a public health facility (51.2%). Utilization of ANC and institutional delivery among women from rural areas was mainly at public health facilities, with overall lower rates of utilization compared to women from urban areas. The wealthiest women also used ANC services more in private health facilities than in the public facilities (51.2% vs. 27.0%), while public health facilities were mainly used for delivery (46.9% in public facilities vs. 14.6% in private facilities). Women from poorer households used more public health facilities (1st to 3rd quintile), while the poorest household used less private health facilities overall. Women from poorer households had low utilization of institutional deliveries (10.7%) compared to women from wealthier households (61.5%).

**Table 3:** Utilization of antenatal care and institutional delivery by household characteristics

	Antenatal care (Mean ± Std. Err.)			Institutional delivery (Mean ± Std. Err.)		
	All	Public	Private	All	Public	Private
Residence						
Urban	0.854 ± 0.0049	0.304 ± 0.0064	0.556 ± 0.0068	0.652 ± 0.0064	0.512 ± 0.0070	0.139 ± 0.0049
Rural	0.570 ± 0.0044	0.341 ± 0.0043	0.208 ± 0.0034	0.245 ± 0.0036	0.214 ± 0.0035	0.031 ± 0.0014
t value	43.05***	−4.85***	45.59***	55.28***	38.16***	21.18***
Education						
No education	0.597 ± 0.0041	0.335 ± 0.0039	0.246 ± 0.0033	0.284 ± 0.0035	0.240 ± 0.0034	0.044 ± 0.0015
Some education	0.824 ± 0.0092	0.329 ± 0.0107	0.483 ± 0.0109	0.612 ± 0.0110	0.503 ± 0.0111	0.110 ± 0.0062
t value	−22.41***	0.49	−20.36***	−27.82***	−22.35***	−10.24***
Wealth^∆^						
1st quintile	0.488 ± 0.0096	0.349 ± 0.0091	0.105 ± 0.0057	0.107 ± 0.0057	0.098 ± 0.0055	0.009 ± 0.0017
2nd quintile	0.610 ± 0.0099	0.406 ± 0.0099	0.176 ± 0.0071	0.193 ± 0.0077	0.180 ± 0.0075	0.014 ± 0.0022
3rd quintile	0.617 ± 0.0092	0.347 ± 0.0091	0.255 ± 0.0080	0.310 ± 0.0086	0.275 ± 0.0083	0.034 ± 0.0032
4th quintile	0.621 ± 0.0084	0.303 ± 0.0081	0.310 ± 0.0077	0.373 ± 0.0083	0.321 ± 0.0081	0.051 ± 0.0037
5th quintile	0.777 ± 0.0066	0.270 ± 0.0068	0.512 ± 0.0075	0.615 ± 0.0074	0.469 ± 0.0076	0.146 ± 0.0051
*F* value	179.77***	38.42***	531.10***	847.08***	462.12***	193.09***
Age						
15–19	0.666 ± 0.0186	0.377 ± 0.0186	0.262 ± 0.0155	0.403 ± 0.0181	0.345 ± 0.0174	0.057 ± 0.0079
20–24	0.655 ± 0.0082	0.342 ± 0.0083	0.302 ± 0.0073	0.360 ± 0.0078	0.308 ± 0.0076	0.052 ± 0.0033
25–29	0.628 ± 0.0078	0.327 ± 0.0076	0.285 ± 0.0068	0.323 ± 0.0071	0.270 ± 0.0068	0.052 ± 0.0031
30–34	0.629 ± 0.0096	0.336 ± 0.0093	0.270 ± 0.0080	0.312 ± 0.0085	0.260 ± 0.0081	0.053 ± 0.0037
35–39	0.596 ± 0.0106	0.327 ± 0.0103	0.258 ± 0.0088	0.297 ± 0.0093	0.249 ± 0.0089	0.048 ± 0.0037
40–44	0.566 ± 0.0150	0.327 ± 0.0142	0.223 ± 0.0114	0.255 ± 0.0122	0.205 ± 0.0113	0.050 ± 0.0054
45–49	0.536 ± 0.0240	0.298 ± 0.0226	0.219 ± 0.0187	0.257 ± 0.0202	0.203 ± 0.0188	0.054 ± 0.0093
*F* value	9.01***	1.79	8.04***	15.04***	15.60***	0.33
Total	0.624 ± 0.0037	0.334 ± 0.0037	0.274 ± 0.0030	0.322 ± 0.0032	0.271 ± 0.0032	0.052 ± 0.0015

Same notes as Table 2

**Fig. 1:**
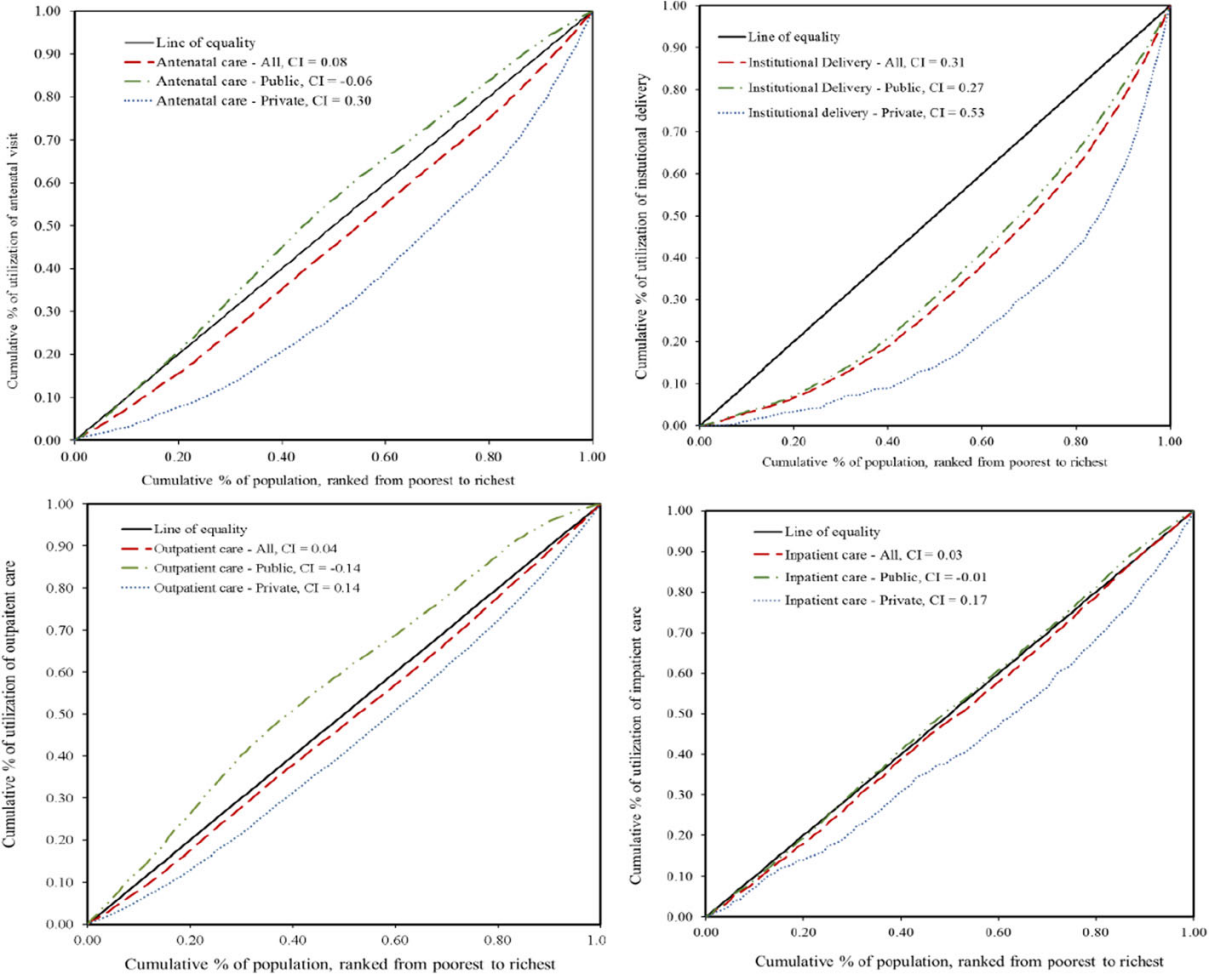
Concentration curves

Table 4 shows the distribution of inequality in health care utilization by service type. The concentration index quantifies the degree of socioeconomic-related inequality in a health variable, such as health care utilization [27]. The concentration indexes show that utilization of ANC, outpatient care, and inpatient care in public health facilities tended to be pro-poor (−0.063, −0.144, −0.011). All services used in private health facilities had positive concentration indexes, indicating greater utilization by the wealthy. Institutional deliveries in public or private health facilities were utilized mainly by the wealthy and were the least pro-poor service provided by public health facilities. The concentration curves are illustrated in Fig. 1. Curves that fall above the line of equality indicate a greater tendency to be pro-poor, whereas curves that fall below the line of equality favor the wealthy. Concentration curves for public health services tended to fall closer to the line of equality compared to the curves for services obtained at private health facilities for outpatient care, inpatient care, ANC, and institutional deliveries.

**Table 4:** Inequality of health care utilization (concentration index)

	Antenatal care			Institutional delivery		
	All	Public	Private	All	Public	Private
Age	−0.001	0.000	−0.001	−0.001	−0.001	0.000
Schooling	0.009	0.001	0.019	0.027	0.027	0.026
Urban	0.047	−0.013	0.136	0.130	0.112	0.229
Residual	0.025	−0.050	0.141	0.158	0.136	0.271
Inequality (total)	0.081	−0.063	0.296	0.314	0.273	0.525
95% CI (total)	(0.074–0.089)	(−0.076–-0.050)	(0.281–0.310)	(0.301–0.327)	(0.259–0.288)	(0.495–0.555)
	Outpatient care			Inpatient care		
	All	Public	Private	All	Public	Private
Age	0.053	0.054	0.053	0.068	0.068	0.068
Male	0.003	0.002	0.003	0.011	0.012	0.009
Schooling	0.007	0.005	0.009	0.022	0.019	0.040
Urban	0.014	−0.066	0.052	−0.020	−0.027	0.017
Residual	−0.035	−0.139	0.021	−0.054	−0.082	0.037
Inequality (total)	0.042	−0.144	0.138	0.027	−0.011	0.171
95% CI (total)	(0.036–0.048)	(−0.155–-0.133)	(0.131–0.146)	(0.013–0.041)	(−0.026–0.005)	(0.134–0.208)

**Fig. 2:**
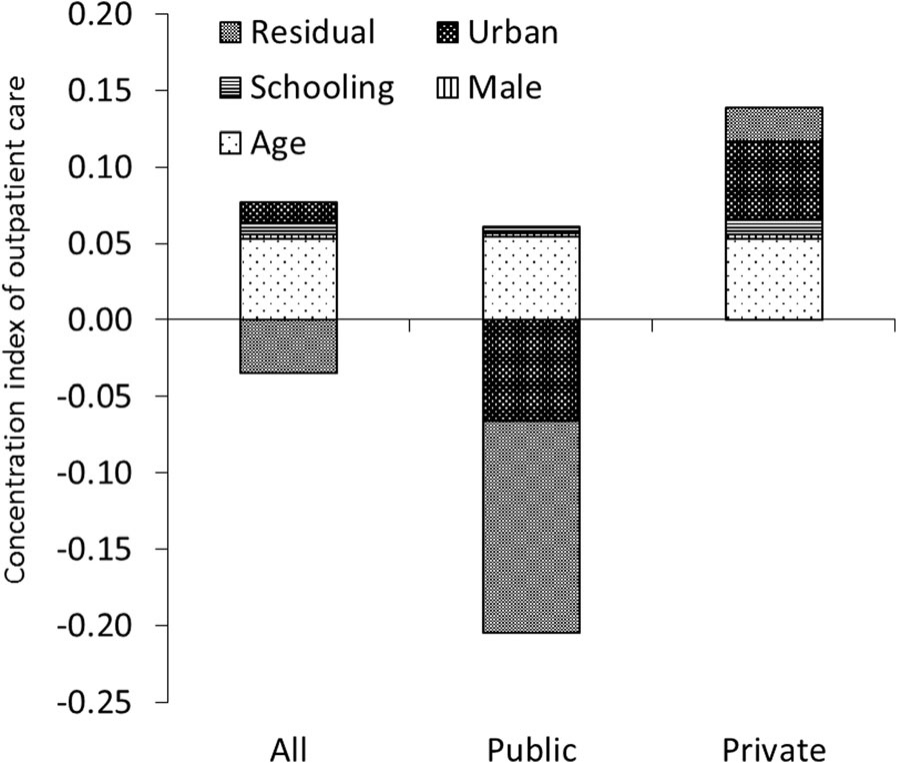
Decomposition of concentration index for outpatient care

**Fig. 3:**
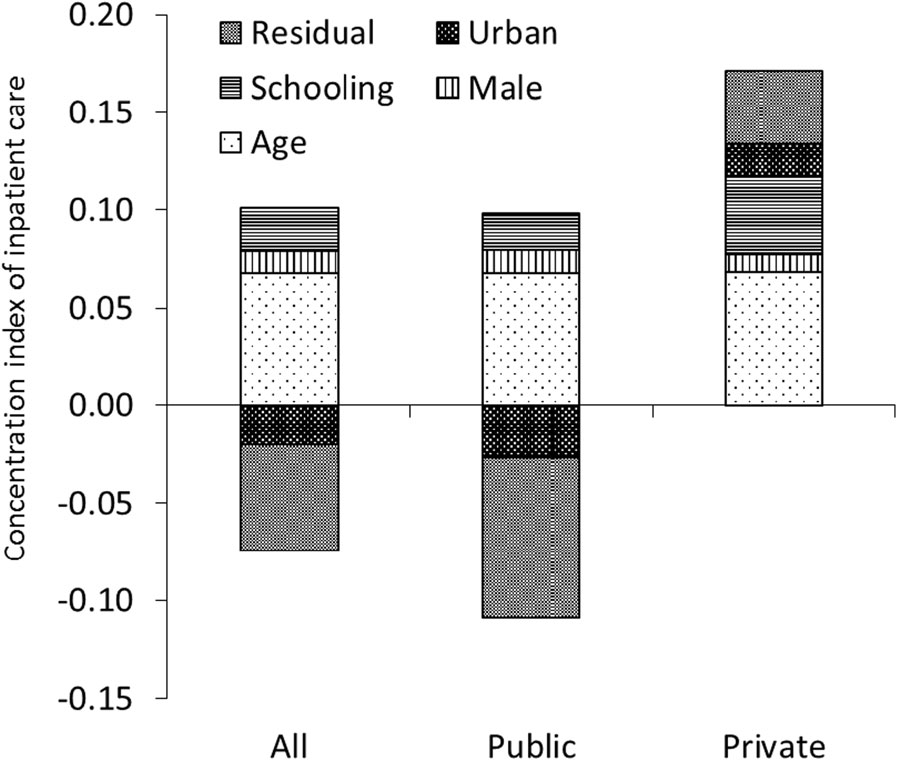
Decomposition of concentration index for inpatient care

**Fig. 4:**
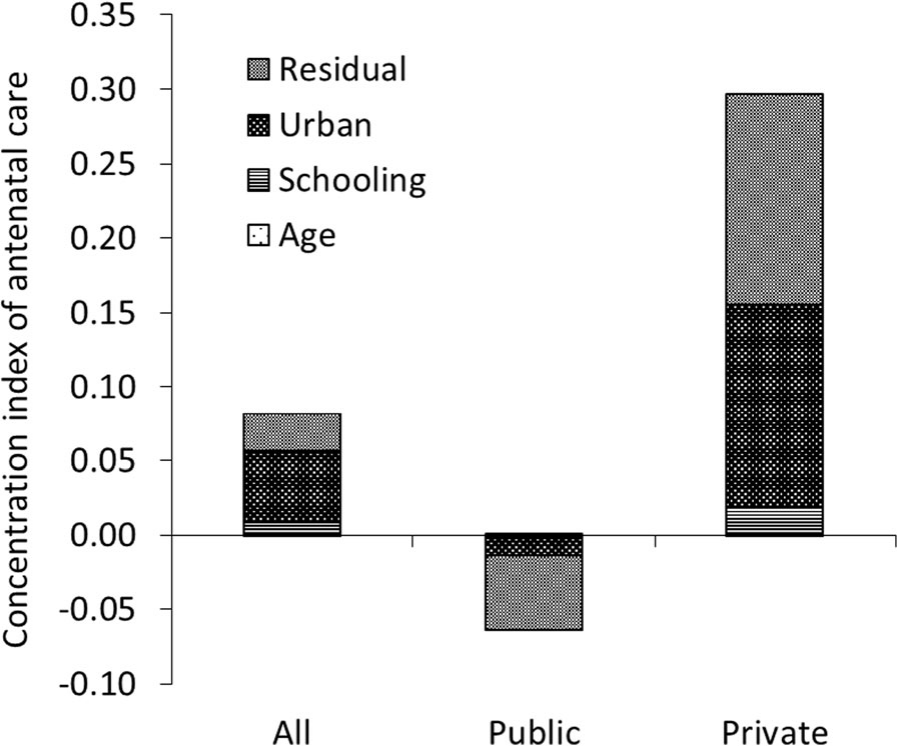
Decomposition for concentration index of ANC

**Fig. 5:**
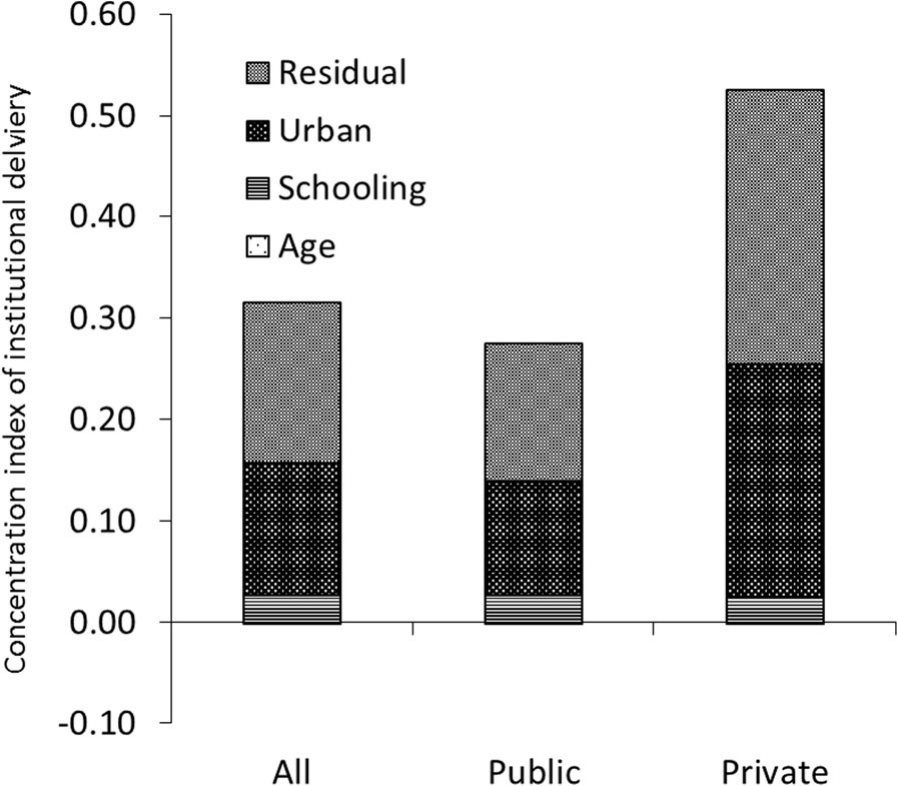
Decomposition for concentration index of institutional delivery

The decomposition of the health concentration index by health determinant (urban, education, age, sex) is also shown in Figs. 2, 3, 4, 5. Geographic residence, or living in urban areas, was the strongest contributor to a positive concentration index for outpatient care, ANC, and institutional delivery in private health facilities, explaining 45.9%, 43.6% and 37.7% of inequality of the utilization of the respective service. Living in urban areas was one of major reasons explaining why the richer using more private health facilities. In addition, living in urban areas explained 41% of inequality of institutional delivery in public facilities. On the other hand, living in rural areas contributes to more pro-poor concentration indexes for public health facilities providing outpatient and ANC services, explaining 21% and 46% of inequality of outpatient care and ANC visits respectively. Age, education, and sex contributed to a bulk of the inequality disfavoring the poor in utilization of outpatient and inpatient services. There was a significant portion of the inequality that could not be explained by age, education, residence and gender.

## Discussion

This study compares the distribution of health service utilization by wealth quintile, residence, and other characteristics, as well as the concentration index between public and private health facilities in Afghanistan. The results support other similar study findings that have shown that overall, the public health system, mainly the BPHS and EPHS, has played a significant role in providing equitable health services for households in Afghanistan [19, 28]. The poorer households were found to use more outpatient services than the wealthy in public health facilities. Poorer women also used more ANC services than the wealthy at public health facilities. Wealthy and poor households had similar levels of utilization of inpatient services in public health facilities.

Poorer women, however, have lower rates in the use of institutional deliveries overall, particularly at public health facilities, compared to the wealthy. The overall institutional delivery rate in Afghanistan is low with less than 50% of women delivering at a health facility with a skilled birth attendant. This is indicative of the large gap between the rich and poor in access to and utilization of maternal health services and a central factor contributing to the high rates of maternal mortality and morbidity in the country [29–31]. This gap requires pro-poor targeted approaches to improve utilization of life-saving maternal health services. Mechanisms for improving utilization of maternal health services include introducing a health equity fund for the poor to support additional costs to accessing health services such as transportation [32, 33]. A pilot study removing user fees at primary health facilities in 2006 showed large increases in utilization of services, though this was not sustained over time likely due to issues of quality of health care services [34]. Another study found that community health funds were feasible in the Afghanistan context but implementation challenges would need to be strongly considered in the design and process for a successful program [35]. Furthermore, multiple financing strategies would be needed to improve access to and quality of health services.

While poorer households used public health services more often, the proportion of public facilities used for outpatient visits remains half of that of private facilities. Public health services through the BPHS and EPHS are free of charge according to the Afghan constitution and health law, however, utilization remains low particularly for ANC services compared to private health facilities [16]. Despite substantial international donor investment in the health sector, utilization of some key maternal health services remains low, suggesting that barriers other than financial constraints should also be addressed. Better understanding of the private sector’s role in increasing equitable coverage of quality maternal health services is needed. Globally among low-income countries, public health services are often used more for antenatal care, however, there is a growing prevalence of private sector as a source for ANC and other maternal health services [36]. Given the considerable share of patients using private facilities and the gap of coverage of BPHS and EPHS to reach more of the population in Afghanistan, policy reforms should take into consideration private health facilities, for example, through public-private partnerships (PPP) in the financing and delivery of health services. Some studies show the favorable impact of PPP in improving quality and coverage of care in some developing countries [37–39]. Afghanistan has put PPP as one of their priorities in health care reform, and will need to learn lessons of PPP in other countries to accelerate its pace in implementing PPP in the health sector.

We also found that differences in utilization of health services across wealth status and between rural and urban residence reinforce poor health among the poorest. For maternal health services such as institutional delivery and use of antenatal care, residence is a major factor explaining inequality of utilization among the population. Other factors that are associated with both social economic status and utilization of health services, such as availability of transportation and accessibility of health related information, may also contribute to the inequality. The impact of residence on inequality of utilization of health services may have to do with the availability of health facilities. Besides the difficulty to avail efficient transportation to get to health facilities, rural areas often tend to have fewer health facilities than urban areas, which also limits the ability of rural populations to seek care. The need to tackle such inequalities within the health system and ensure equitable access to health services has been recognized by the Ministry of Public Health (MoPH) and its partners in the MoPH 2011 five-year Strategic Plan. To increase equitable access to health services, the government focused on the expansion of BPHS and hospital services for reproductive, maternal, neonatal, and child health; increased coverage of prevention and treatment of communicable diseases and malnutrition; and improved quality of care, particularly for rural and hard-to-reach communities. Yet in order to reach the most poor, appropriate allocation of resources and pro-poor policies are needed to meet the poorest population’s actual health needs. Despite the unpredictable and volatile changes in the country’s political, economic, and security situation, reasonable progress has been in the health of the population, though challenges in equitable access to health care remain [28]. From the equity perspective, it is likely that the most insecure areas have greater challenges in accessing health care compared to those areas with reasonable security, and as suggested by Akseer et al., targeted and innovative strategies may be needed to overcome these challenges, including training local workers who are already embedded in these areas [28].

Governments have generally been responsible for providing basic services for the poor. Therefore, public subsidies to reduce poverty and improve health should disproportionately benefit the poor; however studies have shown that for developing countries, this is not often practiced. Health outcomes for the poor are worse than those who are better off, due to various reasons including out-of-pocket payments, expenses related to travel to obtain treatment, limited access to quality health services, and poor health seeking behaviors [40–42]. The growing emphasis on UHC encourages policy makers to ensure that public resources are ‘pro-poor’ or targeting and benefiting the lowest socio-economic groups [43].

There are several limitations to be acknowledged in this analysis. Firstly, the AMS mortality results have been widely discussed to have serious flaws due to the implausible improvements in maternal and child mortality ratios [44]. However, at the time of analysis, the AMS was the most recent data source available. The Multiple Indicator Cluster Survey data from a similar period, later showed similar utilization estimates of maternal and child health services. More recently, the 2015 Afghanistan Demographic and Health Survey showed even more improvements in mortality outcomes. Furthermore, the AMS was the only survey that collected maternal and child health service utilization as well as outpatient and inpatient utilization of the general population in both public and private health facilities. This highlights the challenges of collecting robust and reliable data in insecure countries, so while we recognize the data limitations, we believe these data can still be used to understand the health system and inform policies and programs as best possible. Secondly, while we assess the utilization of private health services, we have little understanding of the availability of the private health services and their scope due to the lack of relevant national data. Although we documented substantial inequality in the use of private health facilities for all four services included in this study, it is not clear whether the availability of private services plays an important role in explaining the inequality. Thirdly, we did not assess the quality of health services and how differences in quality of public compared to private health services affect their utilization. Thus the pro-poor tendency of use of antenatal care, outpatient care and inpatient care in public facilities does not necessarily lead to better health outcomes among the poor. Future studies should consider incorporating quality measures to better understand patient decision-making, equity in access, and their relation to inequality of health outcomes. Lastly, we included age and gender as “need” variables in the inequity analysis. However, not all the gender and age differences of utilization of services are justifiable, some of these difference should also be considered as inequity. Unfortunately, we are not able to discern them.

## Conclusions

Many resources have become available on equity analysis and the need to consider health equity in health systems reforms to truly achieve “health for all” [45–47]. Over the past few years, international institutions have rallied to develop guidance and tools on measuring and monitoring health inequities in countries [48, 49]. The Countdown to 2015 Equity Analysis Group has resulted in several equity trend analyses of countries in changes in coverage of maternal, newborn, and child survival interventions [50–52]. Equity analyses have been conducted across various themes including maternal health [53, 54], immunization coverage [55], and child mortality [56]. A recent equity analysis on contracting mechanisms in Afghanistan recommended linking equity goals to performance bonus provided to service providers as an effective strategy for reducing the inequities [14].

A key factor to institutionalizing equity-oriented health systems reform is increasing country capacity to conduct equity-related research and analysis. While health research capacity has improved in low-income countries, capacity remains weak and should be strengthened [57, 58]. We conducted this analysis using the World Bank ADePT Software which is a tool that streamlines and standardizes complex statistical analysis for more applied economic research by country institutions and governments [49]. Tools such as ADePT have allowed the Afghanistan MoPH to better understand the value and application of equity analysis, use previously collected data for more in-depth secondary analysis not commonly conducted, and understand its policy implications for improving the health for all.

Tracking progress against key health indicators for often marginalized or more vulnerable subpopulations is important to understanding real health system successes and gains. Evaluating health interventions at the national level for effectiveness is not enough. Equity gains for the poorest populations contribute to significant overall gains in health improvement and should be sustained over time [59]. Equity-oriented achievements should be equally lauded by country governments and the international community in order to promote more inclusive health systems.

## Abbreviations

AMS: Afghanistan mortality survey
ANC: Antenatal care
BPHS: Basic package of health services
CIs: Concentration indices
EPHS: Essential package of hospital services
MoPH: The Ministry of Public Health
PPP: Public-private partnerships
SDGs: Sustainable development goals
UHC: Universal health coverage
